# Retraction: Nestin suppression attenuates invasive potential of endometrial cancer cells by downregulating TGF-β signaling pathway

**DOI:** 10.18632/oncotarget.28763

**Published:** 2025-08-19

**Authors:** Amber A. Bokhari, Tabari M. Baker, Batsukh Dorjbal, Sana Waheed, Christopher M. Zahn, Chad A. Hamilton, G. Larry Maxwell, Viqar Syed

**Affiliations:** ^1^Uniformed Services University, Department of Obstetrics and Gynecology, Bethesda, MD 20814, USA; ^2^American College of Obstetricians and Gynecologists, Washington, DC 20024, USA; ^3^Women’s Health Integrated Research Center at Inova Health System, Department of Defense Gynecologic Cancer Center of Excellence, Annandale, VA 22003, USA; ^4^John P. Murtha Cancer Center at Water Reed National Military Medical Center, Bethesda, MD 20889, USA; ^5^Inova Fairfax Hospital, Department of Obstetrics and Gynecology, Falls Church, VA 22042, USA; ^6^Uniformed Services University, Department of Molecular and Cell Biology, Bethesda, MD 20814, USA

**This article has been retracted:** This decision follows an investigation by Oncotarget journal regarding concerns raised on PubPeer. Our image forensics analysis revealed several instances of external overlaps, duplications, and reuse of images from unrelated experiments. Specifically, our findings are as follows:

Figure 6B western blot of Nestin Knockdown Tumors lanes “TGF-beta R2” were duplicated from Figure 4B ChAT lanes 4–8 of an unrelated 2012 article [1].

Figure 6B western blot of Nesting Expressing Tumors lanes “TGF-beta3” are duplicates of modified lanes MMP-9 in Figure 4B of an unrelated paper [2], and the “TGF-beta1” western blot is a duplicate of the Bcl2 western blot in Figure 7B of an unrelated paper [3].

Figure 3B Ishikawa P21 lanes were reused as HEC-2B N-WASP blot, and Figure 5B western blot for MMP-9 was reused as HEC-1B-N-WASP in Figure 7B in the 2017 paper by the same authors [4], which has recently been retracted.

Additionally, Uniformed Services University (USU), where the work was conducted, informed the journal about their internal research misconduct investigation. Their findings include: “(1) Plagiarizing and falsified by reusing, rotating and relabeling lanes 1–3 MMP-9 of Figure 4B in PLoS One. 2013; 8:e76519 as Nesting Expressing Tumors TGF-beta3 in Figure 6B of Oncotarget 2016; 7:69733–69749. (2) Plagiarized and relabeled lanes 1–5 of ChAT lanes in Figure 4B of PLoS One; 7:e51826 as Nestin Knockdown Tumors TGF-betaR2 lanes in Oncotarget 2016; 7:69733–69749. (3) Plagiarized and falsified by reusing and relabeling Ishikawa P21 lanes of Figure 3B in Oncotarget 2016; 7:69733–69749 as HEC-2B N-WASP (55 kDa) lanes in Figure 7B of Oncotarget 2017; 8:113583–113598.” The USU has requested the retraction of the paper. They also noted: “Although we have not received a response from Dr. Syed; she has not been an employee of the USU since January 2023, all other co-authors support this retraction request. Notably, no other co-author on the publication was implicated in the research misconduct investigation.”

Considering both the USU findings and the results of our Scientific Integrity office investigation, the editorial decision has been made to retract the article. All authors and USU were informed of the retraction.

Original article: Oncotarget. 2016; 7:69733–69748. 69733-69748. https://doi.org/10.18632/oncotarget.11947
